# *Artemisia scoparia* Enhances Adipocyte Development and Endocrine Function *In Vitro* and Enhances Insulin Action *In Vivo*

**DOI:** 10.1371/journal.pone.0098897

**Published:** 2014-06-10

**Authors:** Allison J. Richard, Scott Fuller, Veaceslav Fedorcenco, Robbie Beyl, Thomas P. Burris, Randall Mynatt, David M. Ribnicky, Jacqueline M. Stephens

**Affiliations:** 1 Pennington Biomedical Research Center, Louisiana State University, Baton Rouge, Louisiana, United States of America; 2 Department of Pharmacological & Physiological Science, Saint Louis University, St. Louis, Missouri, United States of America; 3 Department of Plant Biology and Pathology, Rutgers University, New Brunswick, New Jersey, United States of America; Pennington Biomedical Research Center, United States of America

## Abstract

**Background:**

Failure of adipocytes to expand during periods of energy excess can result in undesirable metabolic consequences such as ectopic fat accumulation and insulin resistance. Blinded screening studies have indicated that *Artemisia scoparia* (SCO) extracts can enhance adipocyte differentiation and lipid accumulation in cultured adipocytes. The present study tested the hypothesis that SCO treatment modulates fat cell development and function *in vitro* and insulin sensitivity in adipose tissue *in vivo*.

**Methods:**

*In vitro* experiments utilized a Gal4-PPARγ ligand binding domain (LBD) fusion protein-luciferase reporter assay to examine PPARγ activation. To investigate the ability of SCO to modulate adipogenesis and mature fat cell function in 3T3-L1 cells, neutral lipid accumulation, gene expression, and protein secretion were measured by Oil Red O staining, qRT-PCR, and immunoblotting, respectively. For the *in vivo* experiments, diet-induced obese (DIO) C57BL/6J mice were fed a high-fat diet (HFD) or HFD containing 1% w/w SCO for four weeks. Body weight and composition, food intake, and fasting glucose and insulin levels were measured. Phospho-activation and expression of insulin-sensitizing proteins in epididymal adipose tissue (eWAT) were measured by immunoblotting.

**Results:**

Ethanolic extracts of *A. scoparia* significantly activated the PPARγ LBD and enhanced lipid accumulation in differentiating 3T3-L1 cells. SCO increased the transcription of several PPARγ target genes in differentiating 3T3-L1 cells and rescued the negative effects of tumor necrosis factor α on production and secretion of adiponectin and monocyte chemoattractant protein-1 in fully differentiated fat cells. DIO mice treated with SCO had elevated adiponectin levels and increased phosphorylation of AMPKα in eWAT when compared to control mice. In SCO-treated mice, these changes were also associated with decreased fasting insulin and glucose levels.

**Conclusion:**

SCO has metabolically beneficial effects on adipocytes *in vitro* and adipose tissue *in vivo,* highlighting its potential as a metabolically favorable botanical supplement.

## Introduction

Adipocytes have long been recognized as storage sites for excess energy. A growing body of evidence has demonstrated that adipose tissue can also act as an endocrine organ and functions as an important regulator of whole-body energy homeostasis. The endocrine actions of adipocytes can regulate physiological processes, including whole-body insulin sensitivity, glucose uptake, and feeding behavior [1], [2]. Obesity is the primary disease of adipocytes and is a risk factor for the development of type 2 diabetes mellitus (T2DM), cardiovascular disease, and certain cancers. During periods of energy excess, obesity can result from either adipocyte hypertrophy (increased cell size) or adipocyte hyperplasia (increased cell number) or a combination of the two. Since mature fat cells do not proliferate, adipogenesis accompanies hyperplasia of progenitor cells [3]. In recent years, the inhibition of adipogenesis was thought to be an attractive therapeutic modality for the attenuation or prevention of obesity. However, evidence has since emerged demonstrating that disruption of adipocyte differentiation and/or expansion is maladaptive and leads to ectopic fat accumulation and metabolic dysfunction, including insulin resistance and T2DM [4], [5]. Furthermore, increased adipocyte turnover due to an elevated rate of cell death can be associated with obesity [6]. These findings favor an alternative hypothesis that adipose tissue expansion associated with increased adipogenesis results in adipocytes with beneficial endocrine functions that contribute to an improved metabolic status during obesity.

Plant-derived nutritional supplements have long been used by many cultures to promote health and resilience and to prevent or treat disease. Plant extracts have also been used in the development of many drugs, including metformin, a widely used T2DM drug derived from French lilac. In an effort to identify botanical modulators of fat cell development and function, we screened a large variety of botanical extracts. Results from this blinded screening effort led to the identification of an ethanolic extract of *Artemisia scoparia* (SCO) that enhanced adipocyte differentiation and lipid accumulation. Several *Artemisia* species have been used in traditional medicine in East Asia, and have been shown to lower blood glucose [7], [8], combat obesity [9], [10], and prevent T2DM [8], [11] in rodent models of obesity and metabolic dysfunction.

Since our initial findings indicated that SCO promotes adipogenesis, we then examined the ability of SCO to modulate fat cell development and function *in vitro* and insulin sensitivity in adipose tissue *in vivo*. Our novel results demonstrate that SCO activates the ligand binding domain (LBD) of peroxisome proliferator activated receptor gamma (PPARγ), a critical transcriptional regulator of fat cell differentiation [12]. Our results also indicate that SCO increases the transcription of several PPARγ target genes in differentiating 3T3-L1 cells and rescues the negative effects of tumor necrosis factor α (TNFα) on adipocyte secretion of adiponectin (ADPN) and monocyte chemoattractant protein-1 (MCP-1). In diet-induced obese (DIO) mice fed a high fat diet (HFD) or a HFD containing SCO for 4 weeks, SCO increased the activation and expression of insulin-sensitizing proteins in adipose tissue. These changes were associated with decreased fasting serum insulin and glucose levels in SCO-treated mice. Overall, the data demonstrate that SCO has metabolically beneficial effects on adipocytes *in vitro* and adipose tissue *in vivo,* and indicate its potential as a metabolically favorable botanical supplement.

## Materials and Methods

### Preparation, source, and characterization of the extracts

*Artemisia scoparia* Waldst. & Kit ethanolic extracts were prepared at Rutgers University. Briefly, the herb was greenhouse-grown from seed and periodically harvested at the flowering stage, then freeze-dried and stored at −20°C. The dried herb was extracted in 80% ethanol (1∶20 w/v) at 50°C with sonication for 1 hour followed by shaking at room temperature for 24 h. The solid material was removed by centrifugation at 3000 g and the solvent was subsequently removed by evaporation. For *in vitro* and cell culture experiments, the dried extracts were solubilized in 100% dimethyl sulfoxide (DMSO) at a concentration that was 1000-fold higher than experimental concentrations and then diluted into the media. For animal studies, the extracts were mixed with the HFD, and the resulting mixture was pelleted.

### PPARγ LBD activation assay

Botanical extracts were assessed for their ability to modulate the activity of the PPARγ LBD using a previously described Gal4 DNA binding domain-PPARγ LBD fusion protein co-transfection assay system [13]–[15]. All extracts were tested at 50 µg/ml and rosiglitazone (Rosi) was used as a positive control. DMSO was used as a vehicle control.

### Cell culture

Murine 3T3-L1 preadipocytes, purchased directly from Dr. Green's laboratory [16], were plated and grown to 2 days post-confluence in Dulbecco's modified Eagle's medium (DMEM) containing 10% bovine serum. Medium was changed every 48–72 hours. Cells were induced to differentiate using a standard MDI induction protocol [17]. Mature adipocytes were maintained in DMEM supplemented with 10% FBS until utilized for experimentation. DMEM was purchased from Sigma-Aldrich (St. Louis, MO). Bovine and fetal bovine sera were purchased from HyClone (Thermo Scientific, Logan, UT). For adipogenesis assays, the botanical extracts were added at the time of MDI induction and at each media change until the cells were stained with Oil Red O (ORO) or harvested for gene expression analysis. Fully differentiated adipocytes were used for the TNFα experiments. To initiate treatment (day 0), the media was changed to DMEM supplemented with 5% calf serum. The cells were maintained in this media for the duration of the experiment with fresh media added on day 2. Beginning on day 0, the mature adipocytes were treated with 25 µg/mL SCO, 1 µM rosiglitazone (Rosi), or DMSO vehicle daily. TNFα (1 nM) was also added as indicated on days 1 and 3. On day 4, 1 mL of conditioned media was collected from each well and mixed with 1mM phenylmethylsulfonyl fluoride (PMSF). The remaining media was aspirated, and then cell monolayers were collected for gene expression analysis.

### Oil Red O staining

An ORO stock was prepared as previously described [18]. Cell monolayers were aspirated, rinsed with PBS, fixed in 10% formaldehyde in PBS, and rinsed under tap water. The remaining water was aspirated, and the cells were incubated for 1 h in the working ORO solution (0.3% in isopropanol). Following incubation, stain aspiration, and rinsing, cells were examined by microscopy and scanned to produce the figures in this manuscript.

### Animals and diets

Twenty 16-week old male C57BL/6J-60% DIO mice were purchased from Jackson Laboratories (Bar Harbor, ME). Upon arrival to PBRC, mice were housed two per cage and placed into quarantine for one week. After quarantine, mice were randomized by body weight into 2 groups: HFD or HFD + SCO, and then singly housed under constant temperature and humidity (21±2°C with humidity 65–75%) and a 12∶12 h light-dark cycle. Mice had *ad libitum* access to assigned diets and water for the duration of the experimental period. Mice had access to HFD containing 20 kcal% protein, 20 kcal% carbohydrate, and 60 kcal% fat (D12492; Research Diets, Inc., New Brunswick, NJ) or HFD +1% w/w SCO. Food intake was measured weekly for the duration of the experiment. All animal studies were performed with approval from the Pennington Biomedical Research Center Institutional Animal Care and Use Committee (Permit #665P).

### Animal Study Procedures

On day 0 and days 7, 14, 21, and 28 of the experimental period, mice were weighed and non-fasting body composition was measured by NMR (Bruckner Minispec). Submandibular blood collection was performed on each mouse following a 4-hour fast at the beginning (day 0; baseline measurements) and end of the 4-week study period (day 27) to assess fasting levels of serum glucose and insulin. Fasting glucose levels were measured using a YSI glucose analyzer (YSI Life Sciences, Yellow Springs, OH). Fasting insulin levels were determined by a Crystal Chem rat/mouse insulin enzyme-linked immunosorbent (ELISA) kit (Crystal Chem, Downers Grove, IL). Mice were overnight fasted on day 28 and sacrificed on day 29. Ten minutes prior to sacrifice, animals were injected with saline or 1 U/kg insulin (Humulin R, Eli Lilly, Indianapolis, IN) and then euthanized via cervical dislocation and decapitation. The epididymal white adipose tissue depot (eWAT) was collected and immediately frozen in liquid nitrogen.

### Determination of homeostasis model assessment of insulin resistance

The homeostasis model assessment of insulin resistance (HOMA-IR) was calculated using glucose and insulin concentrations obtained after a 4-hour fast, using the following formula: fasting glucose (mg/dl) × fasting insulin (µU/ml)/405 [19], [20].

### Gene expression analysis

Total RNA was isolated from cell monolayers with the RNeasy mini kit (Qiagen). RNA concentrations were measured using a NanoDrop ND-1000 UV-Vis Spectrophotometer. Reverse transcription (RT) was performed using 400–500 ng of RNA and the RT^2^ First Strand Kit (Qiagen). An Applied Biosystems 7900HT thermocycler system was used to conduct all real-time qRT-PCR analyses. For gene expression analysis of the adipogenesis assay, cDNA was quantified using a custom RT^2^ Profiler PCR Array and the RT^2^ SYBR Green qPCR Mastermix (Qiagen). The wet-bench validated mouse RT^2^ Primer Assays used in the custom 12×32 (12 genes by 32 samples) array included: adiponectin (PPM05260), fatty acid binding protein 4 (FABP4; PPM04517), fatty acid synthase (FASN; PPM03816), PPARγ (PPM05108), CCAAT/enhancer binding protein (C/EBP), alpha (CEBPα; PPM04674), CEBPβ (PPM03505), CEBPδ (PPM04676), Sterol regulatory element binding transcription factor 1 (SREBF1; PPM05094), Solute carrier family 2 (facilitated glucose transporter), member 4 (GLUT4; PPM04166), Glyceraldehyde-3-phosphate dehydrogenase (GAPDH; PPM02946), and Peptidyl prolyl isomerase H (PPIH; PPM03699). GAPDH and PPIH were included as housekeeping genes, and all data were normalized against PPIH as the endogenous control. Data were analyzed using the ΔΔC_T_ method via the PCR Array Data Analysis Web Portal (http://www.sabiosciences.com/pcrarraydataanalysis.php). For the gene expression analysis of the TNFα experiments in mature adipocytes, cDNA was quantified using the SYBR supermix reagent (Takara) and the following primers: mAdiponectin forward AAAAGGGCTCAGGATGCTACTG reverse TGGGCAGGATTAAGAGGAACA; mInterleukin-6 (mIL-6) forward TCCTCTCTGCAAGAGACTTCCATCC reverse AAGCCTCCGACTTGTGAAGTGGT; mMCP-1 forward GCAGAGAGCCAGACGGGAGGA reverse TGGGGCGTTAACTGCATCTGG; mCyclophilin A forward CCACTGTCGCTTTTCGCCGC reverse TGCAAACAGCTCGAAGGAGACGC. Standard curves were used to generate relative expression data, and mCyclophilin A was used as the endogenous control.

### Whole cell extract and tissue preparation for protein analysis

Adipocyte whole cell extracts and eWAT lysates were prepared by either harvesting adipocyte monolayers or homogenizing eWAT in a non-denaturing extraction buffer containing 10 mM Tris, pH 7.4, 150 mM NaCl, 1 mM EGTA, 1 mM EDTA, 1% Triton X-100, 0.5% Igepal CA-630, 1 mM PMSF, 1 µM pepstatin, 50 trypsin inhibitory milliunits of aprotinin, 10 µM leupeptin, 1 mM 1, 10-phenanthroline, and 0.2 mM sodium vanadate. For cultured adipocytes, monolayers were scraped into the lysis buffer, subjected to one freeze-thaw cycle, and passed 3 times through a 20-gauge needle. The lysates or homogenates were then centrifuged at 17,500 g for 10 minutes at 4°C. After removing the floating lipid layer, the protein concentrations of the supernatants were determined by a BCA kit (Thermo Scientific, Rockford, IL) according to the manufacturer's instructions.

### Gel electrophoresis and immunoblotting

Reduced and heat-denatured samples were separated on 10% SDS-polyacrylamide gels (acrylamide from National Diagnostics). For native gel electrophoresis, 5% polyacrylamide gels were prepared without SDS. Additionally, SDS and reducing agents were eliminated from the running and sample loading buffers. Following gel electrophoresis, proteins were transferred to nitrocellulose membranes in transfer buffer containing 25 mM Tris, 192 mM glycine, and 20% methanol. Traditional immunoblotting procedures were followed, and results were visualized with horseradish peroxidase-conjugated secondary antibodies and enhanced chemiluminescence (Thermo Scientific, Rockford, IL). Primary antibodies included: the anti-adiponectin antibody from Thermo Scientific (Rockford, IL); phospho-AMPKα (Thr172), phospho-Akt (Ser473), and MCP-1 antibodies purchased from Cell Signaling Technology, Inc. (Danvers, MA); and anti-MAPK (Erk 1/2) antibody from Santa Cruz Biotechnology, Inc. (Dallas, TX). Anti-rabbit and anti-mouse IgG secondary antibodies were purchased from Jackson ImmunoResearch (West Grove, PA). Optical densities of all protein bands were analyzed using Image Studio Lite software (Licor Biosciences, Lincoln, NE).

### Statistical analyses

Gene expression data for ADPN, MCP-1, and IL-6 and optical density data for phospho-AMPKα (Thr172), phospho-Akt (Ser473), and ADPN were analyzed by independent, two-tailed *t*-tests using GraphPad Prism version 6 (La Jolla, CA). Linear models were obtained using SAS 9.3.

## Results

**Figure 1:**
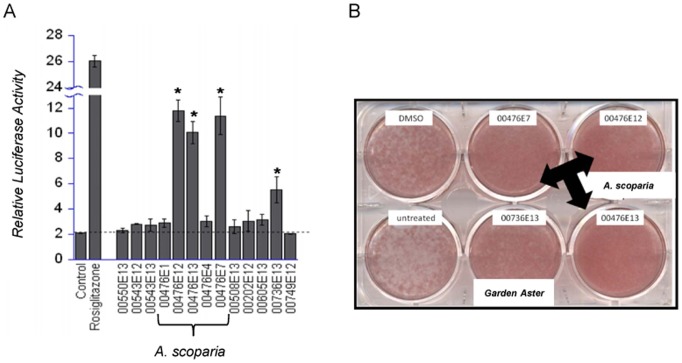
Ethanolic extracts of *A. scoparia* activate the PPARγ LBD and promote adipogenesis *in vitro*. A) HEK293 cells were co-transfected with a Gal4 DNA binding domain – PPARγ LBD fusion protein and a luciferase reporter construct. In a blinded screen, botanical extracts were tested at 50 µg/ml. Untreated (control) and rosiglitazone-treated cells were used as negative and positive controls, respectively. All of the extracts shown were prepared from plants collected in Kyrgyzstan. Assay results are shown for *A. scoparia Walst. & Kit* (00476E1– root, E12– above ground portion of plant, E13– entire plant, E4– stem, and E7– leaf), *Artemisia aschurbajewii C.Winkl.* (00550E13), *Artemisia macrocephala Jacquem. ex Besser* (00543E12– above ground, E13 entire plant), *Artemisia vulgaris L.* (00508E13), *Aster altaicus Willd.* (00202E12), *Aster vvedenskyi Bondarenko* (00605E13), *Aster dumosus L.* (00736E13), and *Crataegus submollis Sarg.* (00749E12). Data are presented as mean ± SEM (n = 3); * denotes p<0.05. B) Murine 3T3-L1 preadipocytes were induced to differentiate using the typical MDI induction cocktail containing 50 µg/ml of the indicated extracts. Cell monolayers were subjected to Oil Red O staining 96 hours after the induction of adipogenesis.

We screened over 400 botanical extracts for their ability to activate PPARγ and modulate adipogenesis in 3T3-L1 cells. As shown in Figure 1A, extracts prepared from *A. scoparia* significantly activated the PPARγ LBD 5–6 fold relative to the untreated control. The well-known synthetic PPARγ agonist and insulin sensitizer, rosiglitazone, was examined as positive control. Rosiglitazone stimulated the PPARγ LBD and increased the luciferase reporter activity by 13-fold over the control values. Multiple SCO extracts (00476) were examined and included preparations from the entire plant (E13), above ground organs (E12), leaves (E7), stem (E4), and root (E1). Only the extracts prepared from the entire plant, above ground organs, and the leaves were capable of significantly activating PPARγ. ORO staining was used to assess the ability of botanical extracts to increase neutral lipid accumulation during fat cell differentiation and served as an indicator of their ability to promote adipogenesis. Compared to the untreated and vehicle (DMSO) treated wells, the *A. scoparia* extracts prepared from the leaves, above ground organs, and the entire plant increased ORO staining during the adipogenesis of 3T3-L1 cells (Figure 1B). These data demonstrate that the SCO extracts that were capable of substantially activating the PPARγ LBD were also adipogenic. As shown in Figure 1, *Aster dumosus L.* (Garden Aster) was also capable of stimulating the PPARγ LBD and promoting adipogenesis. However, in these studies we focused on investigating the effects of an *A. scoparia* extract prepared from the above ground organs (SCO) on adipocyte development and function in 3T3-L1 cells and DIO mice.

**Figure 2:**
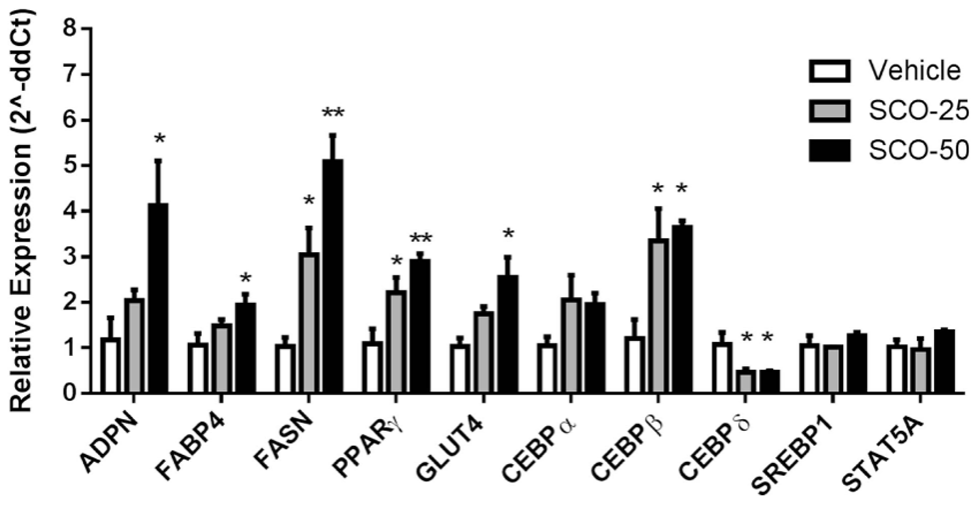
SCO increases the expression of PPARγ target genes associated with adipogenesis in a dose-dependent manner in 3T3-L1 cells. Differentiation of murine 3T3-L1 preadipocytes was induced using the standard MDI induction cocktail containing 25 µg/ml (SCO-25) or 50 µg/ml (SCO-50) of SCO or DMSO vehicle. Cell monolayers were collected on day 5 following administration of the MDI cocktail. RNA was isolated, purified, and subjected to qRT-PCR. Data are presented as mean ± SEM (n = 3) for each gene; * denotes significant difference compared to vehicle (*p*<0.05), ** (*p*<0.01).

To further investigate the ability of SCO to promote adipogenesis, 3T3-L1 preadipocytes were induced to differentiate in the presence of different doses of SCO or DMSO vehicle. The cells were harvested on day 5 after initiating differentiation to examine gene expression. This analysis revealed that SCO substantially upregulated the expression of adipogenic transcription factors, C/EBPs α and β. Moreover, SCO significantly induced expression of PPARγ and several of its target genes in a dose-dependent manner (Figure 2).

After inspecting the data, there appeared to be a linear relationship between SCO dose and gene expression. By treating dose as a continuous measurement, a linear model was used to estimate the effect of dose on gene expression. A strong linear relationship (p<0.01) was shown in PPARγ, GLUT4, and FASN. A linear relationship (p,0.05) was also shown in FABP4, ADPN, and CEBPs β and δ.

**Figure 3:**
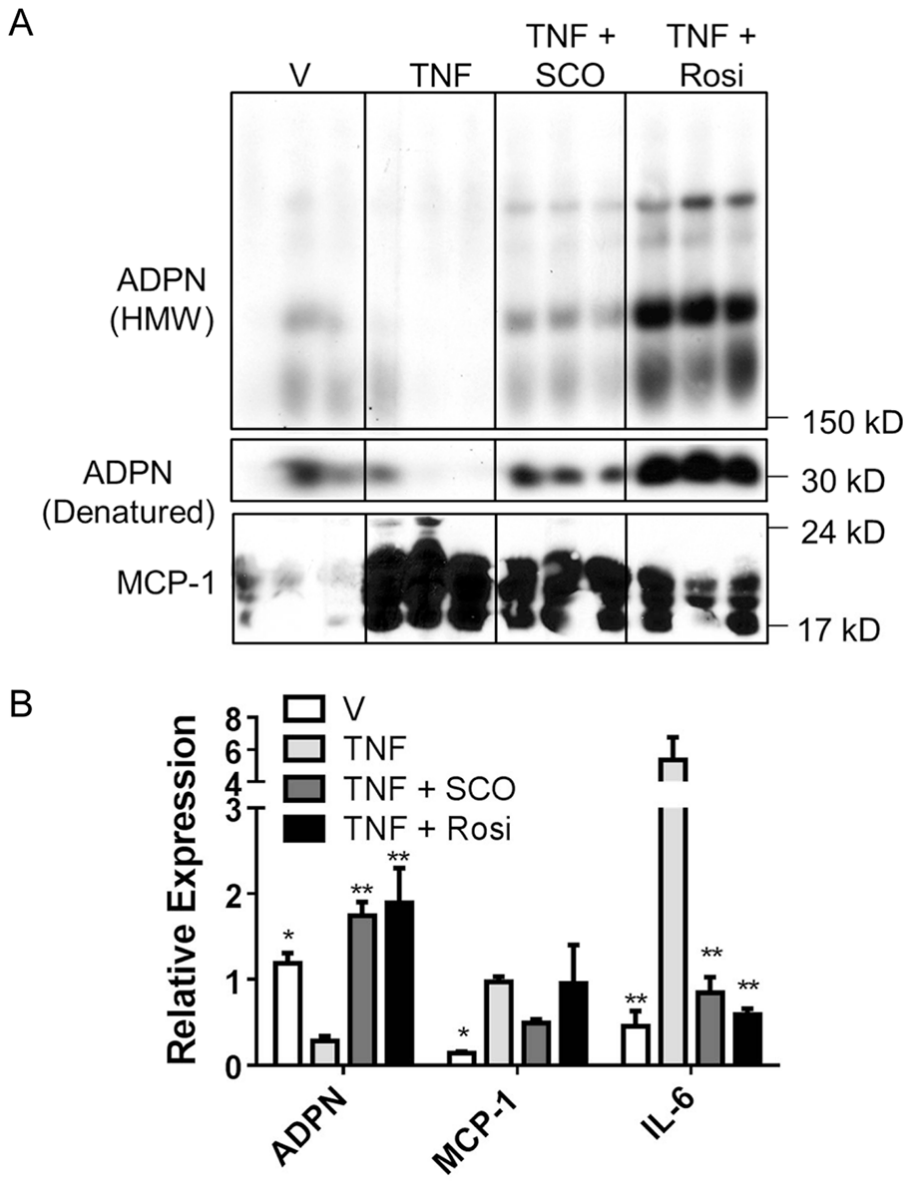
SCO attenuates TNFα-induced effects on adipokine secretion in 3T3-L1 adipocytes. Fully differentiated 3T3-L1 adipocytes were treated for 4 days (treatment began on day 0) in triplicate on six-well plates. Cells were treated with 25 µg/mL SCO, 1 µM rosiglitazone (Rosi), or DMSO vehicle daily. TNFα (1 nM) was also added as indicated on days 1 and 3. On day 4, conditioned media and cell monolayers were collected, respectively, for protein and gene expression analysis. A) Conditioned media samples (250 µg total protein/sample) were separated using SDS-PAGE, transferred to nitrocellulose, and subjected to immunoblot analysis. B) Purified RNA was subjected to qRT-PCR analysis to examine gene expression. Data are presented as mean ± SEM (n = 3). For each gene, * denotes significant difference compared to TNF (*p*<0.05), ** (*p*<0.01).

TNFα is a pro-inflammatory cytokine that has been implicated in insulin resistance and has been shown to inhibit ADPN production [21]–[23]. MCP-1 is a cytokine that recruits monocytes to sites of inflammation and has been shown to contribute to pathologies associated with metabolic syndrome [24]. We used immunoblot analysis to evaluate the effect of SCO on ADPN and MCP-1 secretion from cultured, fully differentiated murine adipocytes that were also exposed to TNFα (Figure 3A). Our results demonstrated that TNFα decreased ADPN secretion and increased MCP-1 secretion from mature adipocytes. These TNFα-induced changes in adipokine secretion were attenuated by SCO treatment (Figure 3A). Analysis of mRNA isolated from the same adipocytes used for the experiments shown in Figure 3A revealed that SCO treatment restored the TNFα-induced downregulation of ADPN expression, and attenuated the TNFα-induced upregulation of MCP-1 and IL-6, another pro-inflammatory cytokine (Figure 3B). Rosiglitazone was used as a positive control in these experiments (Figures 3A and 3B).

**Figure 4:**
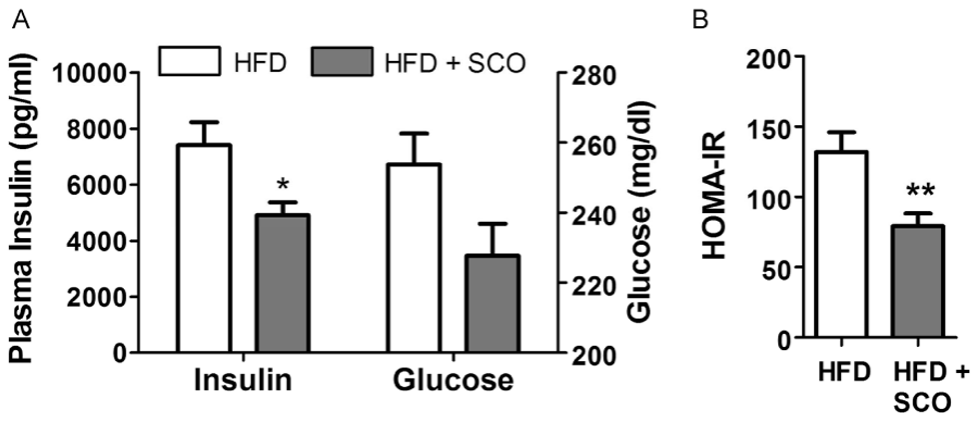
SCO decreases insulin and glucose levels *in vivo*. DIO C57BL**/**6J mice were fed a HFD or HFD + SCO for 4 weeks. A) Following a 4-hour fast, serum insulin and glucose levels were measured. * denotes significant difference relative to HFD control (p<0.02). B) HOMA-IR was calculated from fasting insulin and glucose levels. ** denotes p<0.01. Data are presented as mean ± SEM (n = 9–10).

For our *in vivo* experiments, we fed mice HFD or HFD + SCO for 4 weeks and assessed the effect of SCO on serum insulin and glucose (Figure 4A). The SCO-treated mice had significantly lower serum insulin concentrations compared to controls (*p*<0.02). Analysis of serum glucose revealed that the treated mice had lower glucose levels than controls but the difference did not reach statistical significance (p = 0.0551). However, insulin resistance in the SCO-treated mice was significantly lower compared to controls, as determined by HOMA-IR quantitative assessment (Figure 4B). Results for body weight, body composition, and food intake did not differ between the two groups at the end of the 4-week study period (Table 1).

**Table 1:** Food intake, body weight, and body composition in mice at end of study.

			Body Composition		
Group	Food intake/day	Body weight	FM	FFM	Fluid
	(g)				
HFD	2.85±0.31	47.62±1.02	15.91±1.00	25.12±0.48	4.84±0.15
HFD + SCO	3.06±0.28	45.99±1.08	15.71±1.11	24.44±0.42	4.86±0.09

**Figure 5:**
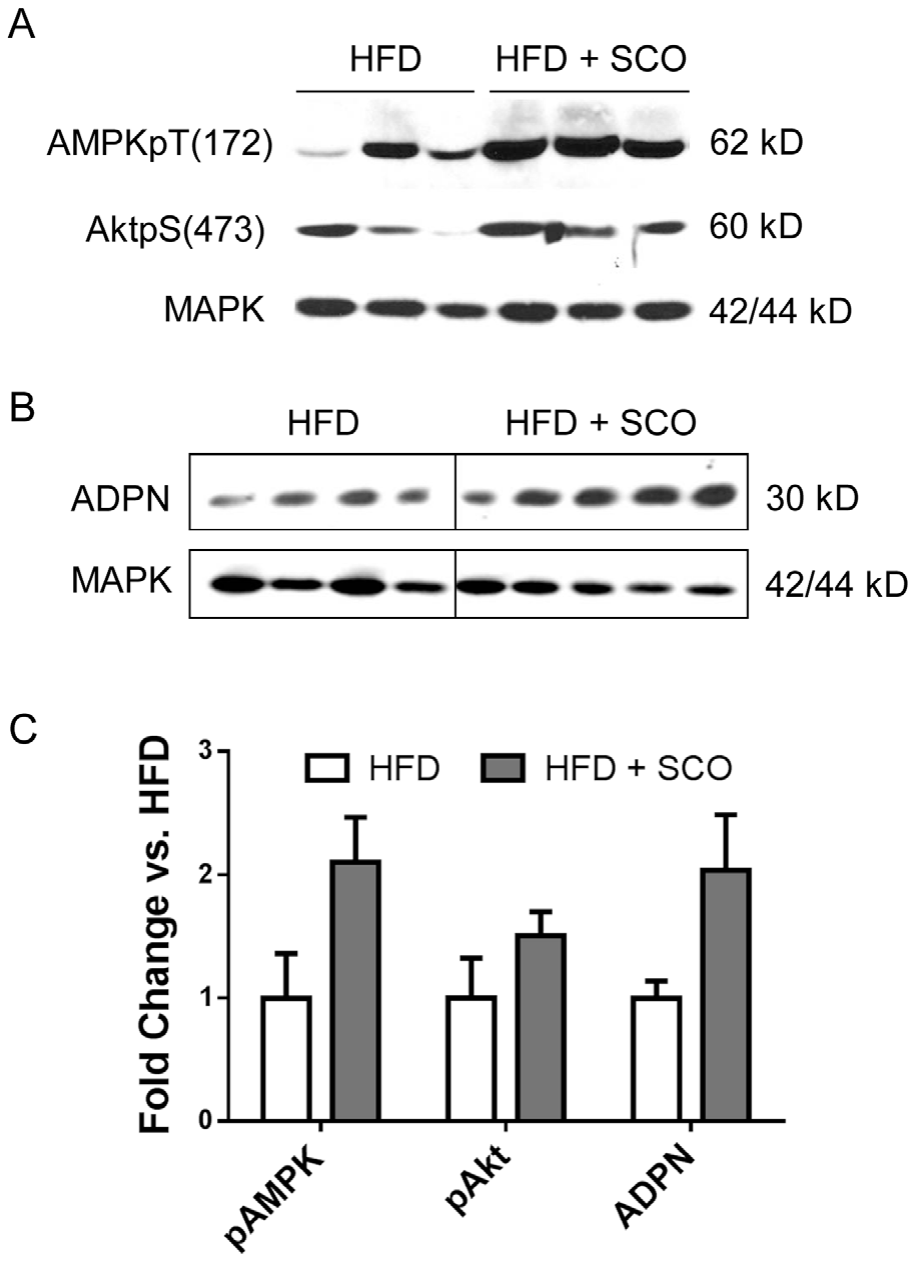
SCO enhances adiponectin expression and AMPK activation in adipose tissue. DIO C57BL/6J mice were fed HFD or HFD + SCO for 4 weeks. Ten minutes prior to sacrifice, mice were injected with insulin. Protein homogenates prepared from eWAT were separated by SDS-PAGE and analyzed by immunoblot analysis. Representative immunoblots for AMPK and Akt (A) and adiponectin (B) are shown. C) Band intensities were quantified using Image Studio Lite software and normalized against the respective MAPK optical densities. Fold change relative to HFD alone for each protein was calculated, and mean ± SEM (n = 4–5) is graphed.

AMP-activated protein kinase (AMPK) is an enzyme that plays a critical role in energy homeostasis in a variety of cells and is activated by adiponectin [25], [26]. Akt plays a central role in several critical cellular responses and is integral to insulin signaling [27]. We assessed the ability of SCO to modulate these proteins in eWAT collected from control or SCO-treated mice. As shown in Figure 5, immunoblot analysis revealed that phosphorylation of AMPK (pT172) was increased in SCO-treated mice as compared to the untreated controls, but this difference did not reach statistical significance (p = 0.068). We also observed a trend toward increased ADPN levels in the eWAT of treated mice compared to controls (p = 0.083), while SCO-treatment did not substantially modulate phosphorylation of Akt (pS473).

## Discussion

The search for effective preventive or therapeutic interventions to combat obesity and T2DM is critical. There is a time-honored tradition of using botanical products in complementary medicine. However, few studies examine biological mechanisms that mediate botanical activity. In our laboratory, results from blinded screening studies examining the effects of botanicals on adipocyte differentiation suggested that *A. scoparia* extracts warranted further investigation. In the present study, we examined the effects of *A. scoparia* on several factors involved in adipocyte development and insulin signaling. The results of our experiments support the hypothesis that *A. scoparia* modulates biological factors relevant to obesity and T2DM and promotes the development of an advantageous metabolic phenotype.

We examined the effects of several *A. scoparia* extracts on PPARγ LBD activation and adipocyte development in murine 3T3-L1 cells. Our results demonstrated that extracts prepared from the entire plant, above ground organs, and leaves significantly activated the PPARγ LBD and promoted adipogenesis as judged by lipid accumulation. These data indicate that the pro-adipogenic bioactive components reside in the leaves and possibly flowers (not individually tested), but are not present in the stems or roots of *A. scoparia*.

PPARγ is a transcription factor that functions a master transcriptional regulator of adipogenesis [28]. Thiazolidinediones (TZDs), a class of anti-diabetic drugs that are potent activators of PPARγ, also modulate adipocyte development and function. The potent insulin-sensitizing activities of TZDs have been effective in diminishing the progression from impaired glucose tolerance to T2DM [29], [30]. However, undesirable side effects have led to recent decreases in the use of TZDs as therapeutics [31]–[33]. Future studies will be performed to assess whether SCO has any of the undesirable effects of TZDs. Currently, non-traditional PPARγ activators that may have anti-diabetic activity are being developed [34], [35]. We predict that botanicals, like SCO, may be a natural source of PPARγ activators that are less potent, but retain anti-diabetic actions.

Our studies demonstrate that SCO increases PPARγ's transcriptional activity, as indicated by its ability to activate the LBD of PPARγ (Figure 1A) and by its induction of several PPARγ target genes (Figure 2). Notably, SCO increased the gene expression of ADPN, FABP4, FASN, and GLUT4, which are all PPARγ target genes, in a clearly dose-dependent pattern. While SCO obviously activates PPARγ, further experiments are needed to determine whether these effects are direct or indirect. SCO may contain a bioactive component that is a direct ligand of PPARγ, but also it is equally possible that SCO-treatment activates PPARγ indirectly in cultured cells by increasing the production of PPARγ ligands or modulating post-translational modifications that control PPARγ activity. Overall, these results provide evidence that SCO promotes adipocyte development *in vitro* and warrant closer investigation of its possible effects in promoting a favorable metabolic status.

Chronic inflammation is a hallmark of obesity and insulin resistance and is highly associated with macrophage infiltration into adipose tissue [36]. TNFα, a pro-inflammatory cytokine that plays a role in adipose tissue inflammation, inhibits the production of adiponectin, an adipocyte-derived hormone that can increase insulin sensitivity and glucose uptake [26], [37]. MCP-1 also is involved in the inflammatory response. Our results demonstrated that SCO attenuated the TNFα-induced increase in MCP-1 secretion and partially reversed the inhibition of adiponectin secretion in response to TNFα treatment, but these effects were not as robust as those seen with rosiglitazone (Figure 3A). The ability of SCO to attenuate the negative effects of TNFα on adipokine secretion was associated with similar responses on the gene expression level (Figure 3B), suggesting that the ability of SCO to modulate adipokine secretion in the presence of TNFα is dependent on transcriptional responses. SCO also blunted the TNFα-mediated increase in IL-6, which is another pro-inflammatory cytokine that has been shown to induce insulin resistance in adipocytes [38], [39]. These results provide evidence that SCO favorably modulates the secretory function of fat cells by attenuating the production of inflammatory markers (MCP-1 and IL-6) in adipocytes and rescuing the production of ADPN. Thus, SCO may have potential as a therapeutic in metabolic syndrome, which is strongly associated with chronic low grade inflammation and insulin resistance.

Our *in vivo* experiments demonstrated that treatment of DIO mice with SCO resulted in significantly lower fasting serum insulin levels when compared to controls. We also observed a reduction in fasting serum glucose that did not reach statistical significance (Figure 4A). Moreover, our finding that the HOMA-IR of the SCO-treated mice was significantly lower compared to controls (Figure 4B) is an indicator that SCO attenuated insulin resistance in these animals. We also have data showing that SCO enhanced insulin-stimulated glucose disposal during insulin tolerance tests in mice that had been administered SCO or vehicle via gavage for one week (data not shown). Taken together, these results suggest that SCO enhances insulin action in mice.

Further analysis of our *in vivo* experiment demonstrated that SCO enhanced the activation and expression of insulin-sensitizing proteins in eWAT. The phosphorylation of AMPK was increased in SCO-treated mice. AMPK is a potent integrator of cellular energy status and is activated when the ATP to AMP ratio is low. In addition to regulating energy homeostasis, AMPK regulates several cellular processes, including mitochondrial function and biogenesis, inflammation, oxidative and ER stress, and autophagy [25]. Recent findings indicate that AMPK plays a role in adipose tissue by regulating lipid and carbohydrate metabolism, adipogenesis, and inflammatory pathways [40]. In addition to measuring AMPK phosphorylation, we also examined phosphorylation of Akt, a critical insulin signaling enzyme that also modulates several other key cellular processes, such as apoptosis, proliferation, and cell migration [27].

The finding that SCO treatment enhances AMPK phosphorylation in mouse eWAT provides further evidence that this botanical could promote metabolically favorable adaptations. In rodent muscle, activated AMPK has been shown to directly phosphorylate the insulin receptor, resulting in non-insulin-dependent stimulation of the insulin signaling pathway [41]. Increased AMPK phosphorylation is associated with improved insulin sensitivity and decreased IRS-1 serine phosphorylation, which improves insulin action [42]. However, we did not observe improved insulin signaling in the eWAT of SCO-treated mice relative to the controls, as determined by examining insulin-responsive Akt serine phosphorylation in the eWAT homogenates. Since AMPK also modulates adipose tissue lipid metabolism [43], it is possible that increased AMPK phospho-activation in eWAT results in less lipid release and increased lipid oxidation in fat tissue that could be associated with less ectopic fat accumulation in liver and muscle and enhanced insulin sensitivity and glucose regulation in the whole animal. Further studies examining plasma triglyceride levels and ectopic lipid accumulation in muscle and liver are needed to test this hypothesis.

Although the present study did not assess the effect of SCO on insulin action in liver and skeletal muscle, there is experimental evidence to show that SCO and other related *Artemisia* species increase insulin-stimulated Akt phosphorylation in both of these tissues [44]–[47]. We cannot exclude the possibility that treatment with SCO affected insulin signaling in the liver and skeletal muscle of the treated animals in this study and therefore could partially account for the observed decrease in insulin and glucose.

## Conclusions

The findings from our *in vitro* and *in vivo* studies on the effects of SCO on adipocyte development and function and insulin action indicate its potential as a therapeutic capable of promoting metabolically advantageous changes in the context of obesity/T2DM. The potency of SCO as a PPARγ agonist indicates that further investigation is necessary to determine its efficacy as a complementary therapy in the prevention and/or treatment of T2DM; SCO may have some of the insulin-sensitizing benefits of TZDs without the unfavorable side effects. SCO has positive effects on several biological markers of insulin resistance, inflammation, and adipocyte function. The attenuation of pro-inflammatory proteins, MCP-1 and IL-6, along with the stimulation of adiponectin expression in adipocytes and adipose tissue, demonstrates the viability of SCO as an agent that promotes the maintenance of metabolic health. Results from our studies suggest that *A. scoparia* should be further studied in the search to find metabolically beneficial therapeutics for obesity and T2DM and to understand the mechanism(s) by which botanicals can improve metabolic dysfunction.
